# Supplementary material for: Patterns of Intron Gain and Loss in Fungi
Source: PLoS Biol. 2004 Nov 30;2(12):e422. doi: 10.1371/journal.pbio.0020422 (PMC532390; doi:10.1371/journal.pbio.0020422)
Supplement: Table S1 — Also available at http://genes.mit.edu/NielsenEtAl/. (4.3 MB ZIP). [file pbio.0020422.st001.zip › NielsenEtAl/html/920.html]

AN8002.1.NCU01377.1.MG07440.1.FG06092.1


```
 CLUSTAL W (1.82) Multiple Sequence Alignments - Introns Inserted


Sequence 1: MG07440.1	664 aa
Sequence 2: FG06092.1	616 aa
Sequence 3: NCU01377.1	654 aa
Sequence 4: AN8002.1	756 aa
Alignment Length: 794 aa
Number Identitical Residues: 258 aa
Alignment Score (without introns) 13567


MG07440.1 	-------MEQFLRDWRQDALNKAQYESAIFVGDKLLAITG1DDKDAFWLAQVHFASGSYT
NCU01377.1	MASSQPNMEKFLREWRQDALNKAQYESAIFIGDKLLAMTN1DDNDAFFLAQVHFAAGNYT
FG06092.1 	-------MESFLREWRQDALNKAQYESAIFIGDKLLALTN1DDQDAFWLAQVHFATGNYT
AN8002.1  	-------MENYLRVWRQQAKVRGQYDAAVFIGDKVLALTN1SDEDALWLAEVHFSNNNYT
          	       **.:** ***:*  :.**::*:*:***:**:*. .*:**::**:***: ..**

MG07440.1 	RAQALLSKSDLVSRNPSCRYLSAHCLIKQGQYDEALNVLGDRNPTHLIPTASNKRRARGP
NCU01377.1	RAQALLVKQDLLTSSPSCRYLAAHCLIKQNNFVEALAILGEHTPTHLFATNAKRKTTRGL
FG06092.1 	RAQAFLSSQNLISRNPSCRYLAGHCLIKQSRFDEALSVLGDRNPTHLIANGASNKRKTQP
AN8002.1  	RALAILSRQDLISRSTACRYLAAHCYIKQGQYEQALTVLGDQNPTHLIRSNKSRRKIQHL
          	** *:*  .:*:: ..:****:.** ***..: :** :**::.****: .  ..:     

MG07440.1 	DAGARSKQPSTGFSRSAAAITGAKSSLSLERQHQRDDEATAAAEEEDALVRRYEAGMCYL
NCU01377.1	RG-----------------TTARASSKTPSRHHDQGD----DIPEEDIPSRRYEASMCYL
FG06092.1 	------------------------------RQTRRRD----PVAEEEASNRRYEAAMCFL
AN8002.1  	NG---------------------HSRITLRNAKSRYE----DRDREDAGNIRYEAGMCYL
          	 .                      :  :  .   : :       .*:    ****.**:*

MG07440.1 	RGLCFAKQNAFDRAKECYKDAVRIDVQCFEAFQQLTKNHLMSPDEEWQFLESLGFDSIRV
NCU01377.1	RGLCYAKQNAFDRAKEAYKDALRIDVQCFEAFSQLVKNSLMSPDEEDEFMQSLAFDSIRA
FG06092.1 	RGICYAKQNAFDRAKECYKDAVRIDVQCFEAFQQLMNNNLLSPDEEWQFLESLDFDAIHV
AN8002.1  	RGLCFAKQNAFDRARDCYKDAVRIDVQCFEAFDQLMKNSLMSPAEELEFLESLDFDSITG
          	**:*:*********::.****:**********.** :* *:** ** :*::** **:*  

MG07440.1 	STPGGEGDDDASAEAATFTKMLYSTRISKYRNPDAFQTAAESLSSHYGLANNPDLLLARA
NCU01377.1	P----GDDPDMEDEPADYVHMLYQTQLSKYRNPQAFNTAIESLSTHYGLENNADILLAKA
FG06092.1 	S-----GDASASQQAAEFTKMLYTTRLSKYRNPAAFETAYDSLSTHYHLASNPDLQLARA
AN8002.1  	A------DAPISQEAADFTRMLYTTRLSKYSSPAVLTDATETLSTHYKLAENPDILLSRA
          	.      *   . :.* :.:*** *::*** .* .:  * ::**:** * .*.*: *::*

MG07440.1 	DLLYTQCRYRDALGITGSILDADK---------------YNFSVYPLHLACLHELRMTNV
NCU01377.1	DLLYTQCRFKDALAITESILMDDK---------------YNFAVYPIHLACLYELRKTNV
FG06092.1 	DLLYTQCRYRDALNITNEILQEDK---------------YNFSVYPVHLACLFELKEKNL
AN8002.1  	EALYTQCRFAEALELTSSILSTSRSSLSAQTTAGQNHLGHSPTVYPLHLACLYETGATNA
          	: ******: :** :* .**  .::: ::.:::...  .:. :***:*****.*   .* 

MG07440.1 	LFLVAHDLADHHPEEPCTWLAVGIYYFSIGRVAEARRYFSKASMMDAHFGPAWIGFAHTF
NCU01377.1	LFLIAHELADTHPDEPCTWLAVGIYYFTTGKIADARRYFSKASMMDANFGPAWIGFAHTF
FG06092.1 	LFLIAHDLADSHPEEPCSWLAVGIYYFSIGKIPEARRYFSKASMMDAHFGPAWIGFAHTF
AN8002.1  	LFLLSHTLADHSPEESYTYLAIGVYYLSVAKIAEARRFFSKASLLDPHSAPAWIGFAHTF
          	***::* ***  *:*. ::**:*:**:: .::.:***:*****::*.: .**********

MG07440.1 	AAEGEHDQAISAYSTAARLFTGTHLPQVFLGMQNHAMNNMTVAEEFLKTAYGLCR-----
NCU01377.1	AAEGEHDQAVTAYSTAARLFTGTHLPQVFLGMQNHAMNNMTAAEEFLKTAYSLCK-----
FG06092.1 	AAEGEHDQAISAYSTAARLFMGTHLPQVFLGMQNHALNNMTLAEEFLKTAYGLCK-----
AN8002.1  	AAEGEHDQAIAAYSTAARLFQGSHLPQLFLGMQHLALNNMSLAQEYLCAAYAMSTGTATG
          	*********::********* *:****:*****: *:***: *:*:* :**.:. .:::.

MG07440.1 	-----------------EDPLLLNEMGVVFYHQDRLKDAAAMFRRALDVAEANDGDPQAW
NCU01377.1	-----------------TDPLLLNEMGIVLYHQDRLKEAVKMFDQALAIAEETDSDPHAW
FG06092.1 	-----------------TDPLLLNEMGVVKYHQDKPKEAAQYFTAALKIADDMDSDPSAW
AN8002.1  	TVPSIPSLPSSEMSPLGGDPLVLNELGVVLYHQNHLEGAVDLFRQALGLATSLRCEPGAW
          	: .: .: .::. :. . ***:***:*:* ***:: : *.  *  ** :*     :* **

MG07440.1 	LSARTNLAHALRRQRHFNQALDQFDEVLRLGGKDP-------------------------
NCU01377.1	LGARTNLGHALRRLRKHDEALEEFDQVLRDGGKDA-------------------------
FG06092.1 	LAARTNLGHAFRRLRHFNRALAEFDEVLRQGGKDA-------------------------
AN8002.1  	VATRSNLGHALRRLGRYSAALDEFDECLRIGSSGASLGYSPFLGGSGGNASGVASAGVSG
          	:.:*:**.**:**  :.. ** :**: ** *....: . :.  ..:...::. :::. :.

MG07440.1 	--------AIFCAKGLIHLDQGRPGDAVVVLHEALAISPQDP---------IATELLNKA
NCU01377.1	--------QIFCAKGLIYLDQGRPDEAVRVLHEALAIHPQDP---------IATELLNKA
FG06092.1 	--------AIFSAKGLILMEQNKPEEAVTVLHEALAVNPQDS---------IATELLNKA
AN8002.1  	YEERGLIGSLYTARGLVLLEMNRTLDAVTTLHEAVRVLGASGGGDAAGGAGVAGTLLSRA
          	 .. .  . :: *:**: :: .:. :** .****: :   . ...::..:.:*  **.:*

MG07440.1 	LEDTAGATLLGGEDGDDDDDALGATGPDIAA-----------------FERGLVER--DR
NCU01377.1	LEESAEAGGIGLLGTADADLGGEAGDEAITAS----------------FEQLLGGAARMR
FG06092.1 	LEETI-----------LQDGAAESEAEDLIE-----------------FERMLGQQKHDA
AN8002.1  	LEIWALETRETEAGLSEDGNRAAKSSTRSRDKGKSRAARRRIAADDSYAEQWIDEVTGGV
          	**           .    .            ...: ::    ::..:  *: :       

MG07440.1 	ALREQRQARAAATAAAG---AAGRPVRDKGKGKER--AGTRHRRRE----SLMDMSDIE-
NCU01377.1	LARERGRRAVAETPGQGGVKDKGKGVARGGRRGAA--GGTRLFRGDQEEVDSMELSD---
FG06092.1 	AVKVNGGRRTVGRMEKG---KGRVRRSRRMTVLED--ENDSEEKED----GMMEMTDDE-
AN8002.1  	PTGLDSTNTVDETIEMELDQDAERLLRDSVEHIRGGLRGRREHIHQPLSSPEVEAQQAQP
          	         .         .               .  .      :  .   ::  : ..

MG07440.1 	---------------
NCU01377.1	---------------
FG06092.1 	---------------
AN8002.1  	RSRGTRTARSYQARS
          	 : .: :: : .: :
```
